# The Impact of Heat Waves on Emergency Department Admissions in Charlottesville, Virginia, U.S.A

**DOI:** 10.3390/ijerph15071436

**Published:** 2018-07-07

**Authors:** Robert E. Davis, Wendy M. Novicoff

**Affiliations:** 1 Department of Environmental Sciences, University of Virginia, Charlottesville, VA 22904-4123, USA; 2 Departments of Public Health Sciences and Orthopaedic Surgery, University of Virginia, Charlottesville, VA 22908, USA; wendy@virginia.edu

**Keywords:** heat wave, emergency admissions, heat-related morbidity, apparent temperature, Charlottesville, Virginia

## Abstract

Heat waves have been linked to increases in emergency-related morbidity, but more research is needed on the demographic and disease-specific aspects of these morbidities. Using a case-crossover approach, over 700,000 daily emergency department hospital admissions in Charlottesville, Virginia, U.S.A. from 2005–2016 are compared between warm season heat wave and non-heat wave periods. Heat waves are defined based on the exceedance, for at least three consecutive days, of two apparent temperature thresholds (35 °C and 37 °C) that account for 3 and 6% of the period of record. Total admissions and admissions for whites, blacks, males, females, and 20–49 years old are significantly elevated during heat waves, as are admissions related to a variety of diagnostic categories, including diabetes, pregnancy complications, and injuries and poisoning. Evidence that heat waves raise emergency department admissions across numerous demographic and disease categories suggests that heat exerts comorbidity influences that extend beyond the more well-studied direct relationships such as heat strokes and cardiac arrest.

## 1. Introduction

It has been well known for many decades that days with anomalously high heat and humidity are associated with peaks in human mortality [1,2,3,4,5]. The strongest linkages generally have been found with respect to cardiovascular and respiratory diseases [6,7,8,9,10,11,12]. When extended periods of heat are examined, there is evidence of an additional effect associated with the prolonged exposure from the added thermal stress placed on the body coupled with the lack of respite from cooler conditions [13,14,15,16,17,18].

Considerably less research has been conducted on heat waves and morbidity [10,11,19]. With a warming climate, heat waves, by definition, will become longer and/or more extreme [20,21,22], although predicting the human response to these events is complex [23,24,25]. There is considerable disagreement in the literature on relationships between heat waves and emergency department and/or hospital admissions [10,26]. Many studies find an overall increase in all-cause or non-accidental admissions during or just after heat waves [27,28,29,30,31], whereas others find no increase [32]. Heat waves tend to have a greater impact on the elderly [11,28,29,33], but the impacts may vary over the course of the warm season [11]. Renal and respiratory admissions are commonly linked to heat waves [28,34,35,36], although some studies have identified disease-specific local relationships that have not been replicated in other populations [37,38]. Despite, and perhaps because of this lack of convergence of study results, it is incumbent upon health care providers to attempt to understand the relationships between heat waves and morbidity to optimize care, as well as to provide the best available public health information during higher risk periods. 

Is there an association between heat waves and morbidity? If there is, do heat waves disproportionately impact morbidity in certain disease categories, or are heat wave impacts not disease specific? Based on the availability of a high quality, long-term administrative dataset of emergency department admissions in a humid subtropical mid-latitude city, we examine the relationships between heat waves and admissions across a spectrum of demographic and disease categories. Although many previous studies have examined one or several heat waves in a case study approach, our extensive time period allows us to include a larger number of heat waves than is typical of the published research on this topic. Furthermore, rather than focus on one or a few specific diseases, we investigate the entire suite of disease categories, along with other demographic variables. We thus hope to contribute to the existing heat wave morbidity literature by studying a long time period influenced by multiple heat waves and by examining a wider variety of disease and demographic factors.

## 2. Materials and Methods

Daily emergency department (ED) admissions to the University of Virginia Medical Center, located in Charlottesville, Virginia, U.S.A., are compiled from 2005–2016. These data comprise over 720,000 individual admissions with a daily average of 164 (standard deviation = 21). Although roughly half of the individuals were ultimately admitted to the hospital for at least one night, in this research, no distinction is made between those individuals and patients who were released after their ED visit. Data compiled for each person include age, race/ethnicity, gender, and discharge diagnosis as classified according to 21 broad disease categories identified in the 10th revision of the International Classification of Diseases (ICD-10) [39]. The three most frequent diagnostic categories are injury and poisoning (19.1%), respiratory disease (9.7%) and circulatory disease (9.5%) (Table 1). There were several periods when the discharge diagnoses were incorrectly tabulated—late July and August, 2010 and late June, 2012. Those 58 days were deleted from the analysis.

Weather observations are taken at the Charlottesville, Albemarle County Airport (station code: CHO). Charlottesville is a mid-latitude city (38°N) with a humid but variable warm-season climate. Heat waves occur in Charlottesville, but the climate is not dominated by prolonged periods of high heat and humidity. Extreme heat events in this region have previously been linked to high mortality [23,25] but morbidity has not been explicitly examined. We use air temperature (T) and dew point temperature (T_d_) at 1400 Local Standard Time to compute apparent temperature (AT) using the approximation developed by Kalkstein and Valimont [40]:

AT = −2.654 + 0.994T + 0.0153 T_d_^2^. (1)

AT estimates the combined effect of heat and humidity on the human body by approximating heat transfer for a clothed individual [41]. AT is commonly used in biometeorological research to examine heat impacts because it combines the influence of humidity in addition to ambient temperature into a single measure [9,18,35,42,43,44,45]. Our application does not employ the wind speed correction, which typically has a small impact on the AT value. We chose 2 p.m. local time because it is highly correlated with daily maximum temperature (*r* = 0.98) and previous research has indicated a close correspondence to health responses using hourly afternoon and maximum temperatures [46]. Because dew point temperature is needed for the calculation of AT, it is preferable to use observations from a fixed time of day when both variables are recorded rather than a maximum temperature for which the time of occurrence varies daily. Only 27 days (0.06%) were missing T or T_d_ observations—these missing data were linearly interpolated using observations from the preceding and following days. In only one case were data for two consecutive days missing.

In general, our statistical approach involved first defining heat waves as events that are physically-based phenomena that occur independent of the hypothesized impact, and then determining if emergency admissions were elevated during (and immediately after) heat waves compared to control periods in which elevated heat and humidity were not present. In this time-stratified, case-crossover approach, admissions during heat waves (cases) are compared to prior non-heat waves (controls) using a paired *t*-test with bootstrapped confidence intervals and *p*-values. To account for the uncertain relationship between potential exposure and response, lags are also investigated. Additionally, different heat wave thresholds are used to examine how admissions might be affected by heat of increasing severity. The details follow.

A heat wave is climatologically defined as an extended period of unusually high temperatures. Partly because the definition of a heat wave depends upon the sector it impacts (e.g., agriculture, water resources, human health), there is no consistent and routinely applied definition of a “heat wave” [47,48]. Therefore, a variety of heat wave definitions have been developed, with most of the differences between methods related to the temperature variable used, the temperature thresholds, and the minimum event duration [7,22,47,48,49,50]. Astrom et al. [10] and Li et al. [26] review heat wave and health studies showing the wide variety of approaches used in defining prolonged heat events. The heat wave definition used in this research is consistent with other approaches to this problem in the environmental epidemiological literature [9,18,51,52]. The analysis was restricted to the months of April through September. 

We initially identified three AT thresholds based on the April–September data for our period of record: 35 °C, which is the 89th percentile, 37 °C (95th), and 39 °C (99th). Subsequent analysis determined that the 39 °C heat waves resulted in sample sizes so small that this threshold was not subsequently evaluated.

Heat waves are herein defined as at least three consecutive days in which the given threshold AT was equaled or exceeded with no more than one intervening non-threshold day. A non-threshold day could not begin a heat wave, but two consecutive non-threshold days would always end one. To demonstrate the definition, a hypothetical month was created with 11 days that meet the threshold (Table 2). The first event is a 4-day heat wave because day 7 is a single, non-threshold day. Similarly, a 3-day heat wave would be defined on days 13–15. However, the two-day event on days 25 and 26 would not be defined as a heat wave because a non-threshold day cannot begin a heat wave and two consecutive non-threshold days end it. 

Admissions during heat waves are compared to control periods prior to the onset of the heat waves [11,20,53,54]. This case-crossover approach is an attempt to account for possible confounders that vary as a function of time by comparing heat waves to proximate events that were not impacted by heat. Control periods are defined to begin immediately before the onset of the heat wave and are of the same duration but cannot overlap with another heat wave. Controls are only defined prior to the heat wave rather than afterward because of the potential for lingering heat effects on ED admissions that might extend beyond the climatologically-defined end of the event. By defining controls that are temporally proximate to heat waves, time-varying factors related to admissions, including seasonality and long-term trends, are mitigated. Using the previous example (Table 2), the control for the first heat wave (days 5–9) is simply the previous 4 days (days 1–4). However, the heat wave on days 18–21 was removed from the analysis because the 4-day control would overlap with the end of the heat wave on days 13–15. One advantage of this method, in addition to the simplicity, is that it eliminates the need for extensive data standardization and assumptions about the underlying form of time-varying factors, because proximate time periods are being compared. A comparison of this type of approach to generalized model-based time series approaches to heat wave morbidity and mortality showed both methods produced very similar results [55]. Mean daily morbidity during heat events is compared to the controls using a one-sample, paired t-test with the type I error rate set to 0.05. A one-sample test is used because we hypothesize that heat waves would only increase morbidity—we are not hypothesizing that heat waves provide any protective effect. Confidence intervals and p-values are estimated from bootstrapped samples based on 1000 replications.

One possible complication of this method is that there may not be enough heat waves to provide a random day-of-week sample. Results of a one-way analysis of variance using total daily admissions (not shown) presents strong evidence for a day-of-week artifact, with significantly lower admission rates on weekends. To address this possible bias, a second type of control was defined in which the control was lagged one week prior to the onset of the heat wave. Based on our definitional constraints, it is possible to define one-week lag controls for all but two heat waves using the 35 °C threshold. For these two cases only, the previous definition was applied to define the appropriate control periods.

The health-related impacts of heat tend to be immediate or have short lags [16,23,49,56]—we therefore also examine a one-day lag. Furthermore, because ED admissions are mostly elective, in an effort to account for individual differences in response to a heat event, we apply a three-day centered moving average smoother to the ED data. Thus, we examine both non-smoothed and smoothed (3 day) admissions for lags of zero and one day. In addition to total daily admissions, we evaluate admissions by age, race (black, white), gender (female, male), and for 21 discharge diagnostic categories (Table 1). The 10 age categories are <1 year, 1–4, 5–9, 10–19, 20–29, 30–49, 50–64, 65–74, 75–84, and ≥85 years.

## 3. Results

We identified 28 heat waves based on the 35 °C AT threshold, or 2.2 heat waves per year (Table 3). The longest heat wave lasted 12 days and occurred in June 2015. There were no April heat waves, and only two heat waves occurred in late May (2011, 2012). The warmest average heat wave was a 3-day event in July 2011 with a mean AT of 39.4 °C. The highest AT observed was 41.3 °C during a 9-day heat wave in August 2016. At least one heat wave occurred in every year except for 2009. We likewise identified 15 heat waves using a 37 °C AT threshold (Table 3). The 126 heat wave days (using the lower threshold) and 63 days (using the higher threshold) account for 6% and 3% of the period of record, respectively.

### 3.1. 35 °C Heat Waves

Same-day ED admissions were elevated for the total, male, and white categories, for individuals aged 20–49, for patients with pregnancy complications, and for “other conditions” (Table 4). For a 1-day lag, higher admissions were only evident for people aged 20–49. When a 3-day filter was applied to the dependent variables, statistically significant relationships were found for the same groups as in the unsmoothed analysis, but the results were slightly less significant in most cases. Using a 1-day lag on the smoothed data, statistically significant results were found only for the male, white, age 20–29, and age 30–49 categories. 

These results were calculated using control periods that immediately preceded the heat wave. Because of the relatively limited number of events, it is possible that significant differences could arise from a lack of randomness in the controls. In an attempt to address this issue, a second control was applied using a 7-day lag, which should mitigate against biasing from day-of-week artifacts. Using this alternative control definition, admissions during heat waves were significantly higher for total, female, male, black, white, age 20–29, and age 30–49 admissions (Table 5). With respect to the diagnostic categories, ED admissions were elevated for the endocrine, congenital, and “other conditions” groups, as well as for injury and poisoning. Once again, the only results found using a 1-day lag were for patients aged 30–49. The results for smoothed variables were generally consistent with the unsmoothed, with the primary exception being additional significant relationships for total, white, male, and endocrine admissions with a 1-day lag.

### 3.2. 37 °C Heat Waves

The use of a more stringent threshold effectively reduced the sample size by half. For the unlagged and unsmoothed data, higher admissions only occurred for patients aged 20–29 and those individuals diagnosed with diseases of the nervous system and sense organs or digestive diseases (Table 6). However, with a 1-day lag, in addition to age 20–29, three different diagnostic categories were identified: diseases of the skin, congenital anomalies, and diseases of the ear. For the smoothed data, the only relationships found were for digestive diseases with no lag and for people aged 20–29 with or without a lag.

Using the alternate control definition, the results were consistent for all combinations of lags and smoothers. However, the age 20–29 category was no longer significant with a 1-day lag, and admissions were elevated for patients diagnosed with endocrine and digestive issues (Table 7).

## 4. Discussion

Emergency admissions to the University of Virginia Medical Center are significantly elevated during heat waves in which the apparent temperature exceeds 35 °C for three or more days. In addition to total admissions, elevated ED admissions are found for the white, black, male, female, and age 20–29 and 30–49 subcategories. The consistency of these results across smoother and control definitions suggests that the findings are robust for this AT threshold. These subcategories have relatively high daily sample sizes and should therefore provide fairly stable estimates of heat wave impacts relative to the control days. An increase in overall admissions during heat waves is consistent with other research on this topic [27,28,29,30,31], although this result is not universal [32]. Differences in response by location suggest that infrastructure and adaptation influence morbidity impacts, much like they do with mortality [57]. 

With respect to age, some previous research shows a greater impact among the elderly and young children [28,29,32,33,35,58], whereas other studies show elevated ED visits and admissions for all age groups [59,60,61,62]. There is speculation that the number of people in the elderly group (greater than 75) presenting to emergency rooms might not be as high as expected due to other factors, such as isolation, lack of access to immediate care, and a perception that they are not in danger (even given the presence of multiple comorbidities). Similar to the case with cardiovascular diseases, these elderly patients die quickly (or alone) [63,64,65]. For Charlottesville, the only age response to heat was for individuals between 20 and 49. This could be a sample size issue, as the 20–29 and 30–49 year age categories have the largest total admissions. However, Fuhrmann et al. [11] uncovered a similar response in North Carolina to mid-summer heat waves that they attributed to occupational impacts, since jobs that can involve heat exposure tend to employ younger individuals [66].

Conversely, the daily sample sizes for the various diagnostic subcategories are much smaller and the results should be considered with caution. With respect to injuries and poisoning, there is evidence that the rate of injuries, including work-related mishaps, increases during high heat events and with increasing temperatures in general. Wilson et al. [67] examined the impact of extreme heat event duration on mortality and morbidity in Sydney, Australia and found significantly higher rates of hospital admissions for heat-related injuries. Other studies support these findings [68,69,70]. Violence-related injuries are also elevated during high heat events [71,72,73]. 

Diagnostic Category 18 (“Other Conditions”) is the fourth largest category of ED admissions. Many patients present with milder heat-stress illness symptoms such as edema, syncope, cramps, dehydration, and heat exhaustion; these patients would be classified into Category 18 if they do not have underlying comorbidities and their symptoms do not meet the threshold of acute injury [74]. Other than Category 18, the most robust result for diagnostic categories across analyses is for Category 3 (endocrine, nutritional, and metabolic diseases and immunity disorders). People with diabetes are adversely affected by heat stress because of their reduced ability to dissipate heat and greater tendency for dehydration and other fluid imbalances. ED visits and hospital admissions increase during high heat events for this subgroup [75,76,77,78]. 

Few studies have documented evidence of a relationship between heat and pregnancy complications [79,80,81,82,83]. There is limited evidence that heat exposure can lead to lower birth weights, pre-term births, and higher rates of eclampsia and preeclampsia [79,80,84,85,86], but more research is needed on this issue. We could find no evidence in the literature of a relationship between heat and congenital anomalies (Category 14). Our results indicate that heat waves impact both male and female subgroups. The background literature is inconsistent regarding differences in gender responses to heat. Some studies have found differences by sex [60,87], while others have not [54,88,89]. In New York City, data from 2000–2011 shows a higher rate of ED visits for females than males ages 65 and above, but much higher rates for males between ages 15 to 64 [90]. One systematic review [26] noted that certain heat-related illnesses are more common in males (such as occupational impacts) whereas others are more common in females (e.g., renal diseases). 

Similarly, it is often difficult to separate out racial differences from factors that are often correlated with race, such as housing, access to medical care, occupation, etc. [91]. The strongest relationships between high temperatures and morbidity involve vulnerable populations, such as those with limited access to medical care, urban residents, outdoor laborers, racial and ethnic groups (particularly those with low SES), and people with chronic diseases [27,91,92]. Many of these relationships are particularly complex, as they depend on local characteristics such as availability of air conditioning, building standards, pollution, and other issues related to the built environment. This is especially true for ethic groups with lower socio-economic status, who are more likely to live in high-density urban neighborhoods with minimal green space and a lack of resources to mitigate heat exposure. Higher heat-related morbidity and mortality have been reported among African-Americans, who may have more limited access to immediate medical care and have a higher prevalence of chronic diseases that have been shown to be exacerbated by heat, including kidney disease and diabetes [93,94,95].

We found few significant results based on a one-day lag. The impacts of heat tend to be immediate, so this result is generally consistent with prior research on heat and morbidity [55,96,97,98], although some research shows evidence of heat impacts extending up to one week [28]. It is important to remember that these events are all heat waves that last at least 3 days, so a person affected on day one of the heat wave who goes to the ED on or before day 3 would be included in the non-lagged results. Therefore, the lack of a lagged relationship simply suggests that people are not seeking ED treatment on the day after a given heat wave ends. Because a short-term lag is effectively built into the analysis, longer lags beyond one day were not evaluated.

When shorter and more intense heat waves are examined, as defined by the 37 °C AT threshold, the results were internally inconsistent. For these more intense events, we found neither an overall increase in admissions nor higher admissions in any of the subcategories with larger sample sizes. The only statistically significant results were for specific disease categories. In addition to the relationship between heat and endocrine diseases discussed earlier, the most consistent result across sub-analyses was found for diseases of the digestive system. 

Total admissions is positively correlated with admissions for almost all diagnostic categories, although the Pearson correlation coefficients are generally low (Supplementary Table S1). The strongest correlation, with circulatory disease, is only 0.41. Some studies have found a relationship with cardiovascular diseases and admissions [96], but others have not [35,99,100]. One of the largest studies of high temperatures, mortality, and morbidity in European cities showed that although high temperatures have an impact on respiratory admissions, particularly for the elderly, underlying mechanisms are poorly understood [35,99,100]. That same study showed significant relationships between high temperature and cardiovascular mortality, but did not find a relationship between high temperature and cardiovascular morbidity. The authors hypothesized that differences in admissions and mortality could be due to physiopathologic mechanisms (reduced plasma volumes and increases in blood viscosity due to water loss), but that cardiovascular deaths occur very quickly during hot days in compromised patients, so they do not survive long enough to reach the hospital. They also concluded that respiratory mortality tends to peak later than cardiovascular mortality, which could account for the higher numbers of patients presenting to the hospital with these conditions. These results showing heat-related cardiovascular relationships with mortality but not morbidity were supported by other studies [33,101,102]. Our results suggest that heat waves exert impacts across the entire disease spectrum [103]. If so, this relationship has public health consequences with respect to the net impact of heat waves on human health in general. This lack of specificity associated with heat wave morbidity is consistent with heat-related mortality research showing a stronger relationship to total mortality than to mortality for conditions commonly considered to be heat related [5].

A number of limitations of this study must be considered to properly contextualize the results. This analysis was conducted using data for a single hospital, so the extent to which these findings are generalizable is uncertain. However, many of these findings for Charlottesville are consistent with other heat-morbidity research [27,29,53,54,68,103,104,105]. It is likely that thresholds for heat wave ED admission increases will vary spatially based on the prevailing climate, as these responses tend to be relative rather than absolute [12,57]. Furthermore, although daily admissions for the broader categories (total, gender, race) are reasonably large, for the less common diagnostic codes, admissions are necessarily small, so these results should be considered with caution. We made no distinction between ED visits that resulted in hospital admissions and those for patients who were discharged on the same day. Some research has examined air quality impacts on morbidity [100,106,107]. We were unable to incorporate possible air quality relationships because of the lack of consistent local measurements over our period of record. However, the lack of evidence for admissions impacts in the respiratory and cardiovascular disease categories implies that poor air quality is not a confounding factor. It is possible that other factors not incorporated in this study, such as socioeconomic status and pre-existing conditions, also impact ED admissions. We did not have patient-level details available to us in this research, so these components were beyond the scope of this study. Finally, the use of broadly defined diagnostic categories may potentially mask underlying relationships. For example, Lin et al. [108] found positive associations between high temperatures and COPD, asthma, IHD, and cardiac dysrhythmia but negative relationships with hypertension and heart failure. Similarly, several studies have shown increases in emergency room visits for acute kidney injury as well as exacerbations of previously diagnosed renal disease during high heat events worldwide [32,34,75,109,110,111]. Not surprisingly, hospital admissions and calls for emergency services for patients with renal problems also increase during high heat events [28,77,112,113]. Such a detailed subcategorization would be difficult in our case given the small daily sample size for most of these highly specific diagnoses.

## 5. Conclusions

Daily emergency department (ED) admissions to the University of Virginia Medical Center in Charlottesville, Virginia from 2005–2016 were examined to determine if admissions were higher during heat waves. Heat waves were defined as three or more consecutive days with apparent temperatures exceeding a threshold (35 °C or 37 °C) with no more than one intervening non-exceedance day. Admissions were examined by age, race (black/white), gender (female/male), and for 21 broad disease categories based on the ICD 10 code ascribed to each discharged patient.

Heat waves were associated with increased overall admissions as well as admissions for blacks, whites, males, females, and 20–49 years old. Furthermore, a number of disease-specific relationships were found, including endocrine disorders, digestive diseases, injuries and poisoning, and “other conditions,” many of which have symptoms with pathologies that are exacerbated by heat exposure. Interestingly, two of the main overall contributors to total admissions—circulatory and respiratory diseases—did not exhibit higher heat wave admissions. In total, these results suggest that heat waves are associated with morbidity effects that extend across the entire disease spectrum. The results for stronger heat waves based on a more restrictive threshold were less compelling, probably because of the smaller overall number of heat waves.

Much of the prior research on morbidity and heat has emphasized respiratory and circulatory diseases, given the prevalence of these conditions coupled with clear pathologies associated with hyperthermia. Our results suggest that additional research should be conducted to examine the less common diseases that are not typically assumed to exhibit a heat response. Our research suggests that heat waves are a universal health threat that extends across demographic groups and does not necessarily favor individuals with specific predispositions to extreme heat.

## Figures and Tables

**Table 1:** List of diagnostic categories and their relative frequencies.

Diagnostic Category	Diagnosis	Diagnosis (Short Name) ^1^	Relative Frequency (%) ^2^
1	Infectious and parasitic diseases	Infectious	2.9
2	Neoplasms	Neoplasms	0.9
3	Endocrine, nutritional and metabolic disease and immunity disorders	Endocrine	2.6
4	Diseases of the blood and blood-forming organs	Blood	0.5
5	Mental disorders	Mental	5.7
6	Diseases of the nervous systems and sense organs	Nervous	7.9
7	Diseases of the circulatory system	Circulatory	9.5
8	Diseases of the respiratory system	Respiratory	9.7
9	Diseases of the digestive system	Digestive	8.2
10	Diseases of the genitourinary system	Genitourinary	5.5
11	Complications of pregnancy, childbirth, and the puerperium	Pregnancy	2.0
12	Disease of the skin and subcutaneous tissue	Skin	2.4
13	Diseases of the musculoskeletal system and connective tissue	Musculoskeletal	7.9
14	Congenital anomalies	Congenital	0.1
15	Certain conditions originating in the perinatal period	Perinatal	0.2
16	Symptoms, signs, and ill-defined conditions	Ill-defined	2.1
17	Injury and poisoning	Injuries	19.1
18	Other conditions	Other	9.2
19	External causes of injury and poisoning	External	0.5
20	Diseases of the eye and adnexa	Eye	0.1
21	Diseases of the ear and mastoid process	Ear	0.1

^1^ For convenience, the short name is used throughout the text; ^2^ The sum is less than 100% because the “unclassified” and blank categories are not analyzed.

**Table 2:** Example of heat wave and control definitions for a hypothetical month. An “X” indicates which days meets the related criterion.

Date	AT ≥ 35 °C	Heat Wave Days	Control Days
1			X
2			X
3			X
4			X
5	X	X	
6	X	X	
7		X	
8	X	X	
9			
10			X
11			X
12			X
13	X	X	
14		X	
15	X	X	
16			
17			
18	X	X	
19	X	X	
20	X	X	
21	X	X	
22			
23			
24			
25	X		
26	X		
27			
28			
29			
30			

**Table 3:** Climatic details for the 28 heat waves using a 35 °C apparent temperature (AT) threshold and the 15 heat waves based on a 37 °C AT threshold.

35 °C Heat Waves				37 °C Heat Waves			
Dates	Duration (Days)	Mean AT	Max AT	Dates	Duration (Days)	Mean AT	Max AT
28–30 Jun 2005	3	34.1	35.4				
16–20 Jul 2005	5	35.0	37.5				
25–27 Jul 2005	3	39.1	39.8	25–27 Jul 2005	3	39.1	39.8
11–15 Aug 2005	5	36.9	37.9				
02–04 Jun 2006	3	35.3	36.8				
17–21 Jul 2006	5	36.0	37.4				
30 Jul–04 Aug 2006	6	38.1	40.3	31 Jul–04 Aug 2006	5	38.5	40.3
26–29 Aug 2006	4	35.5	37.0				
03–10 Aug 2007	8	37.0	40.3	07–10 Aug 2007	4	38.7	40.3
06–10 Jun 2008	5	37.1	38.2	06–10 Jun 2008	5	37.1	38.2
30 Jul–01 Aug 2008	3	33.4	35.8				
12–14 Jun 2010	3	37.5	38.2	12–14 Jun 2010	3	37.5	38.2
22–24 Jun 2010	3	38.5	39.0	22–24 Jun 2010	3	38.5	39.0
05–09 Jul 2010	5	37.4	39.2	06–08 Jul 2010	3	38.4	39.2
22–24 Sep 2010	3	36.2	37.0				
				16–18 Jul 2010 ^1^	3	36.5	37.8
30 May–01 Jun 2011	3	38.0	39.0	30 May–01 Jun 2011	3	38.0	39.0
08–10 Jun 2011	3	37.9	39.2				
11–13 Jul 2011	3	39.4	40.2	11–13 Jul 2011	3	39.4	40.2
				20–25 Jul 2011 ^1^	6	39.9	42.6
26–29 May 2012	4	35.3	37.5				
02–05 Aug 2012	4	35.8	36.2				
15–20 Jul 2013	6	37.4	38.8	16–20 Jul 2013	5	37.5	38.8
17–19 Jun 2014	3	36.5	37.6				
31 Aug–2 Sep 2014	3	35.7	35.9				
12–23 Jun 2015	12	34.5	37.5				
28–30 Jul 2015	3	35.6	36.6				
05–08 Jul 2016	4	36.6	37.9				
				23–28 Jul 2016 ^1^	6	38.3	40.8
10–18 Aug 2016	9	38.5	41.3	11–17 Aug 2016	7	39.3	41.3
06–10 Sep 2016	5	37.1	38.6	08–10 Sep 2016	3	38.1	38.6

^1^ It is possible for 37 °C threshold heat waves to not also be 35 °C heat waves because of the requirements that the associated control days not overlap with a prior heat wave.

**Table 4:** Summary of paired *t*-test results for 35 °C AT threshold heat waves. Categories with statistically significant differences between admissions for the heat wave case and control days, bootstrapped significance level (*p*-value), and bootstrapped 5th and 95th percentile confidence intervals about the mean difference in admissions. Tests were run for lags of zero and one day and for unsmoothed and data smoothed using a 3-day centered moving average (MA) filter.

Smoother	Lag	Category	Mean Difference	Significance	5% C.I.	95% C.I.
No	No	Total	6.6	0.003	2.4	10.8
		Male	4.1	0.000	1.8	6.4
		White	4.3	0.006	1.4	7.5
		Age 20–29	2.2	0.016	0.3	3.8
		Age 30–49	3.5	0.001	1.6	5.5
		Pregnancy	0.4	0.034	0.0	0.8
		Other	1.4	0.010	0.4	2.6
	1-day	Age 20–29	1.6	0.045 ^1^	−0.2	3.5
		Age 30–49	3.5	0.005	1.2	5.8
3-day MA	No	Total	4.6	0.010	1.1	8.6
		Male	3.1	0.005	1.1	5.2
		White	3.2	0.011	1.0	5.8
		Age 20–29	1.9	0.006	0.3	3.1
		Age 30–49	3.1	0.001	1.3	4.7
		Pregnancy	0.3	0.046	0.0	0.7
		Other	1.0	0.016	0.2	2.0
	1-day	Male	2.8	0.025	0.1	5.3
		White	2.8	0.044	−0.1	6.0
		Age 20–29	1.7	0.021	0.2	3.3
		Age 30–49	3.0	0.0003	1.1	4.9

^1^ On occasion, guidance regarding statistical significance may disagree between *p*-values and confidence intervals because both are bootstrapped. We use the *p*-values to determine statistical significance.

**Table 5:** Same as in Table 4 except the control is a one-week lag.

Smoother	Lag	Category	Mean Difference	Significance	5% C.I.	95% C.I.
No	No	Total	5.7	0.003	2.2	9.1
		Female	2.6	0.040	−0.3	5.1
		Male	3.0	0.019	0.3	5.6
		Black	2.2	0.025	0.2	4.2
		White	3.6	0.017	0.2	6.7
		Age 20–29	1.4	0.038	−0.1	2.8
		Age 30–49	2.9	0.005	0.8	5.0
		Endocrine	1.1	0.001	0.5	1.7
		Congenital	0.1	0.049	0.0	0.2
		Injuries	1.4	0.046	−0.1	3.0
		Other	1.3	0.028	0.2	2.5
	1-day	Age 30–49	2.9	0.008	0.5	5.1
3-day MA	No	Total	4.8	0.003	1.3	8.2
		Female	2.3	0.050	−0.2	5.0
		Male	2.5	0.028	0.1	5.0
		Black	1.8	0.035	−0.1	3.7
		White	3.2	0.021	0.3	6.1
		Age 20–29	1.3	0.022	0.1	2.5
		Age 30–49	3.1	0.001	1.3	4.7
		Endocrine	0.8	0.003	0.3	1.3
		Congenital	0.1	0.036	0.0	0.2
		Other	1.1	0.014	0.3	2.0
	1-day	Total	4.2	0.045	0.1	9.0
		Male	2.7	0.036	0.0	5.7
		White	3.1	0.042	−0.1	6.3
		Age 30–49	2.5	0.012	0.6	4.5
		Endocrine	0.7	0.020	0.1	1.2

**Table 6:** Summary of paired *t*-test results for 37 °C AT threshold heat waves for all smoother and lag combinations. Categories with statistically significant differences between admissions for the heat wave case and control days, significance level, and bootstrapped 5th and 95th percentile confidence intervals about the mean difference.

Smoother	Lag	Category	Mean Difference	Significance	5% C.I.	95% C.I. ^1^
Unsmoothed	No	Age 20–29	2.3	0.011	0.7	3.9
		Nervous	1.5	0.030	0.1	3.0
		Digestive	1.6	0.021	0.3	2.9
	1 day	Age 20–29	2.2	0.018	0.5	3.9
		Skin	0.6	0.048	0.0	1.3
		Congenital	0.41	0.020	0.0	0.3
		Ear	0.2	0.044	0.0	0.4
3-day MA	No	Age 20–29	2.1	0.013	0.7	3.7
		Digestive	1.4	0.03	0.2	2.6
	1 day	Age 20–29	1.8	0.026	0.3	3.5

^1^ Confidence intervals based on a reduced bootstrap of 959 samples.

**Table 7:** Same as in Table 6 except the control is a one-week lag.

Smoother	Lag	Category	Mean Difference	Significance	5% C.I.	95% C.I.
Unsmoothed	No	Endocrine	1.0	0.030	0.1	1.8
		Digestive	1.9	0.011	0.3	3.4
	1 day	Endocrine	0.8	0.045	−0.2	1.7
		Digestive	1.9	0.024	0.3	3.4
3-day MA	No	Endocrine	0.8	0.040	0.0	1.5
		Digestive	1.7	0.010	0.3	3.0
	1 day	Endocrine	0.8	0.033	0.0	1.5
		Digestive	1.8	0.03	0.4	3.1

## Supplementary Materials

The following are available online at http://www.mdpi.com/1660-4601/15/7/1436/s1, Table S1: Pearson’s correlation coefficient between total admissions and admissions for the various categories over all days and during heat waves only.
