# Supplementary material for: The Impact of Heat Waves on Emergency Department Admissions in Charlottesville, Virginia, U.S.A
Source: Int J Environ Res Public Health. 2018 Jul 7;15(7):1436. doi: 10.3390/ijerph15071436 (PMC6068980; doi:10.3390/ijerph15071436)
Supplement: Supplementary file 1 [file ijerph-15-01436-s001.pdf]

Table S1. Pearson's correlation coefficient between total admissions and admissions for the various categories over all days and during heat waves only. An asterisk indicates a statistically significant correlation at the 0.05 alpha level.

Category	All Days	Heat Waves
Female	0.83*	0.80*
Male	0.79*	0.80*
Black	0.61*	0.58*
White	0.87*	0.83*
Infectious	0.03	0.03
Neoplasms	0.09*	0.11
Endocrine	0.26*	0.22*
Blood	0.07*	0.06
Mental	0.29*	0.24*
Nervous	0.33*	0.19*
Circulatory	0.41*	0.32*
Respiratory	0.39*	0.24*
Digestive	0.35*	0.37*
Genitourinary	0.37*	0.33*
Pregnancy	0.16*	-0.03
Skin	0.27*	0.30*
Musculoskeletal	0.40*	0.43*
Congenital	0.02	0.06
Perinatal	0.02	0.17
Ill-defined	0.10*	0.21*
Injuries	0.37*	0.42*
Other	0.35*	0.18*
External	-0.08*	-0.17*
Eye	0.08*	0.15*
Ear	0.07*	0.15*
